# The Presence of Some Minor *Aspergillus* and *Penicillium* Unregulated Mycotoxins in Main Cereals Cultivated in Albania

**DOI:** 10.3390/molecules29225292

**Published:** 2024-11-09

**Authors:** Dritan Topi, Zamir Damani, Janja Babič, Breda Jakovac-Strajn, Gabrijela Tavčar-Kalcher

**Affiliations:** 1 Institute of Food Safety, Feed and Environment, Veterinary Faculty, University of Ljubljana, Gerbičeva 60, 1000 Ljubljana, Slovenia; janja.babic@vf.uni-lj.si (J.B.); breda.jakovacstrajn@vf.uni-lj.si (B.J.-S.); gabrijela.tavcarkalcher@vf.uni-lj.si (G.T.-K.); 2 Department of Chemistry, Faculty of Natural Sciences, University of Tirana, Blvd. Zogu 1, No. 25/1, 1016 Tirana, Albania; 3 Department of Diagnostics and Rehabilitation, Faculty of Medical Technical Sciences, University of Medicine of Tirana, Kongresi i Manastirit Street, P.O. BOX 1000 Tirana, Albania; zamir.damani@umt.edu.al

**Keywords:** mycophenolic acid, cyclopiazonic acid, roquefortine C, penicillic acid, gliotoxin, maize, wheat, LC-MS/MS, Albania

## Abstract

(1) Background: Food and feed safety legislation does not concern all the mycotoxins generated by *Penicillium* and *Aspergillus* spp. Certain mycotoxins, including mycophenolic acid (MPA), cyclopiazonic acid (CPA), penicillic acid (PA), roquefortine C (ROQ C), and gliotoxin (GLI), are regarded as having lower toxicity levels, and hence are not included in food and feed legislation. It is obvious that xenobiotics, including mycotoxins, exert synergistic harmful health effects on human and animal when exposed through food and feed. (2) Methods: The presence of these substances in maize and wheat grown in Albania across two consecutive harvesting seasons was investigated by liquid chromatography and mass spectrometry (LC-MS/MS). (3) Results: The findings indicated the presence of these mycotoxins in maize grain but not in wheat grain. In the 2014 season, they exhibited a higher contamination incidence than in the 2015 season. The most commonly detected mycotoxin was MPA, followed by CPA and ROQ C toxin, while PA and GLI were not detected. The MPA revealed a concentration range of 72.9–3447 μg/kg, with a mean value of 1064 μg/kg. Mycophenolic acid was detected in the maize samples collected during the 2015 season. (4) Conclusions: These findings suggest that focusing the investigation only on “controlled” mycotoxins will not produce a proper risk assessment and may not adequately address the possible harmful impacts of mycotoxins on human and animal health due to mycotoxins’ co-occurrence.

## 1. Introduction

Cereals are susceptible to contamination by harmful and decay-causing microbes at various phases, including growth, harvest, and storage [1]. Molds, a distinct category, can create a wide range of secondary metabolites called mycotoxins. Food can be contaminated directly, such as mold on the item, or indirectly, when processed foods are manufactured using ingredients that are already infected [2,3]. Out of the many mycotoxins, only around 12 are naturally present in significant amounts and have the potential to be toxic enough to raise concerns over the safety of food and feed. The molds that produce mycotoxins of the most worrisome belong to five taxonomic genera: *Aspergillus*, *Fusarium*, *Penicillium*, *Claviceps*, and *Alternaria* [4,5]. This emphasizes the vital importance of food safety and regulation in preventing mycotoxin contamination and guaranteeing the health and welfare of the general population.

Penicillium mycotoxins, distinguished for their quick development throughout transit and storage, present a significant contamination issue in inadequately kept food and animal feed. The Penicillium genus comprises around 350 species of fungus, which are widely distributed and can produce various mycotoxins and other secondary metabolites that find applications and exert positive effects on human health, e.g., antibiotics. In addition to the distinguished mycotoxins, ochratoxin A, and patulin, the presence of cyclopiazonic acid is worth mentioning, albeit to a lower degree. They have a crucial function in simultaneous exposure to other mycotoxins [2,4,6].

**Figure 1:**
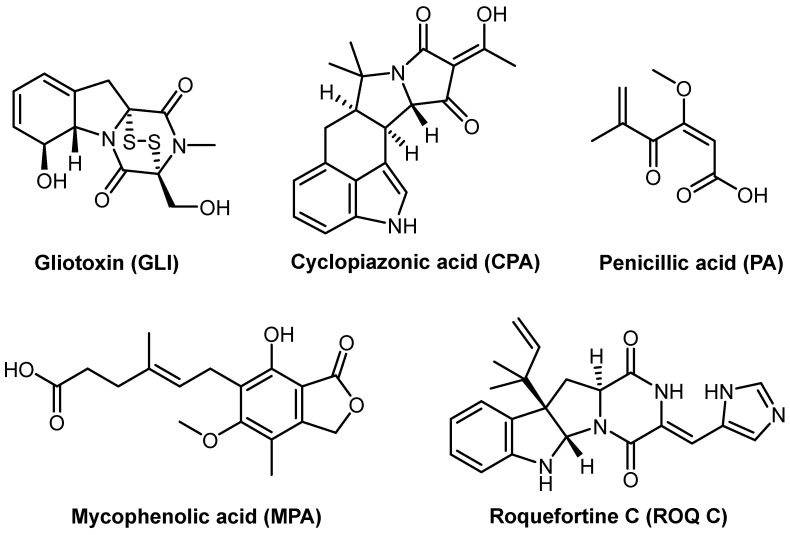
Chemical structures of the studied mycotoxins.

Cyclopiazonic acid (CPA), an indole tetramic acid (Figure 1), was initially identified and described in 1968 [7]. It is synthesized by several species of the *Penicillium* and *Aspergillus* genera [8]. The principal producers within the genus *Penicillium* are *P. commune*, *P. camemberti*, *P. palitans*, *P. dipodomyicola*, and *P. griseofulvum*. From the genus *Aspergillus*, notable species include *Aspergillus flavus*, *A. oryzae*, and *A. tamarii* [4,9]. In addition to colonizing a variety of grains and seeds, these molds can grow on a wide range of foods, including cheese and meat products (Table 1) [10,11]. This growth is attributed to the intake of contaminated feed [7]. While this mycotoxin alone has not received much attention for its hazardous deleterious effects, its presence with aflatoxins in contaminated foods often leads to cumulative impacts [9]. At high levels, it is a potent mycotoxin that may cause localized tissue death in the internal organs of most vertebrates, as well as severe gastrointestinal and neurological diseases [12]. It also acts as a neurotoxic vasodilator, disrupting the muscular contraction–relaxation cycle. This substance is poisonous to many animal species, including rats, pigs, guinea pigs, poultry, and dogs [9].

Penicillic acid (PA) is a mycotoxin derived from polyketides synthesized by many *Penicillium* species and *Aspergillus* genera species (Figure 1) [13]. The chief producers of the *Penicillium* spp. are *P. aurantiogriseum*, *P. cyclopium*, *P. melanoconidium*, and *P. polonicum*, whereas *A. ochraceus* is the primary producer of the *Aspergillus* spp. It is abundantly present with ochratoxin A in high-moisture maize at low temperatures (Table 1) [1,4]. Although its carcinogenic potential is far lower than that of aflatoxins, the concern about its presence in foods stems from its structural resemblance to patulin, a well-known carcinogen. PA’s primary contribution to mycotoxicology is its synergistic toxicity with ochratoxin A [14,15] and its potentially additive or synergistic action with the naphthoquinone hepatotoxins [12]. Penicillic acid contains pharmacological properties that cause vasodilation and have antidiuretic effects. It has a similar behavior to patulin by quickly reacting with dietary molecules that contain sulfhydryl groups, resulting in the formation of harmless substances [1].

Roquefortine C (ROQ C) is a naturally occurring compound that belongs to a family of substances called 2,5-diketopiperazines (Figure 1). Its IUPAC designation is 10b-(1,1-dimethyl-2-propenyl)-3-imidazol-4-methylene-5a,10b,11,11a-tetrahydro-2H-pyrazino-[19,29:1,5]pyrrol[2,3,b]indole-1,4-(3H,6H)-dione [16]. It is synthesized by many types of *Penicillium* fungi, particularly *P. roqueforti*, and other species such as: *P. chrysogenum*, *P. crustosum*, *P. expansum*, *P. hordei*, and *P. griseofulvum* [17]. *P. roqueforti* is a common saprophytic fungus in soil and decaying organic matter. It has the ability to create many mycotoxins, such as patulin, penicillic acid, and mycophenolic acid [18]. Roquefortine C is a significant fungal contaminant commonly found in carbonated drinks, beer, wine, meats, and cheese (Table 1). At low amounts, the presence of this substance in domestic cheeses is deemed “safe for the consumer”. However, at large dosages, it is regarded as a potent neurotoxin [19].

Mycophenolic acid (MPA) is a compound synthesized by some species of the *Penicillium* genus, namely *P. brevicompactum*, *P. roqueforti*, and *P. caneum* [2,4]. MPA, with the IUPAC name 6-(4-hydroxy-6-methoxy-7-methyl-3-oxo-5-phthalanyl)-4-methyl-4-hexenoic acid, is a low-stability organic acid that has immunosuppressive, antiviral, antifungal, antibacterial, and antitumoral properties. Although MPA has modest toxicity to animals, it can nevertheless have a significant impact as an indirect mycotoxin due to its immunosuppressive properties, potentially affecting bacterial and fungal diseases. Corn tainted with harmful fungus metabolites is considered to be the genesis of the epidemic of pellagra disease in the Tyrol region, Italy/Austria, during the beginning of the 20th century [20].

Gliotoxin (GLI) is a mycotoxin containing sulfur in the family of 2,5-diketopiperazines. It is naturally found in the air, soil, and water and is produced by *A. fumigatus* and other species of the *Penicillium* genus [21]. The toxicity processes entail the participation of a disulfide bridge, which seems to create reactive oxygen species by oxidizing the reduced dithiol to its disulfide form [22]. Other authors have found that gliotoxin possesses many immunosuppressive effects [23].

**Table 1:** Some minor *Penicillium* spp. mycotoxins occurrence in plant products, and associated producing species.

Mycotoxin	Agricultural Products	Species	Toxicity	Reference
Cyclopiazonic acid	Long-stored cereals, pasta, meat, and cheese	*P. commune*, *P. camamberti*, *P. palitans*, *P. dipodomyicola*, *P. griseofulvum*	Tissue death in the internal organs in vertebrates. Severe gastrointestinal and neurological diseases. Neurotoxic vasodilation	[12]
			Poisonous to animal species, e.g., pigs, poultry	[9]
Penicillic acid	Cereals, hay, onions, carrots, potatoes	*P. aurantiogriseum*, *P. cyclopium*, *P. melaconidium*, *P. viridicatum*, *P. polonicum*, *P. radicicola*	Synergistic toxicity with ochratoxin A.	[14,15]
			Additive or synergistic action with the naphthoquinone hepatotoxins	[12]
Roquefortine C	Farm silage, cheese, meat products, sugar beet pulp	*P. roqueforti*, *P. carneum*, *P. chrysogenum*, *P. crustosum*, *P. expansum*, *P. paneum*, *P. albocoremium*, *P. allii*, *P. griseofulvum*, *P. hordei*, *P. melanoconidium*, *P. radicicola*, *P. sclerotigenum*, plus another 13 *Penicillium* species	Potent neurotoxin (at large dosages)	[19]
Mycophenolic acid	Cheese, sugar beet pulp	*P. brevicompactum*, *P. roqueforti*, and *P. carneum*	Immunosuppressive effects; pellagra disease	[20]
Gliotoxin	Sugar beet pulp	*A. fumigatus*, *Gliocladium fimbriatum*	Reactive oxygen species producer;	[22]
			Immunosuppressive effects	[23]

Source: [2].

Studying mycotoxins, which can induce many detrimental toxicological consequences in animals and humans, is an essential field that requires more investigation. Animals exposed to this substance over a long period, whether by eating, breathing it in, or absorbing it through their skin, might experience genetic changes, problems with the development of their offspring, miscarriages, and the production of congenital disabilities. Subchronic exposure to some substances in food-producing animals can lead to decreased production, weight loss, a slower development rate, and impaired reproductive function. This emphasizes the importance of conducting further scientific research and gaining better knowledge [1].

We can categorize the observed adverse effects into major and minor mycotoxins. Although minor mycotoxins are not considered dangerous or controlled by regulation, they can have cumulative effects when exposed together with other critical mycotoxins. One example is simultaneous exposure to CPA and ochratoxin A, PA and patulin, ROQ C and patulin, or ROQ C and PA [24]. CPA is a powerful mycotoxin that induces weight loss, diarrhea, convulsions, and mortality in rodents, birds, dogs, and pigs [25]. There have been few recorded cases of animal mycotoxicosis due to the harmless characteristics of CPA [7].

This study examined the simultaneous presence of five mycotoxins (cyclopiazonic acid, penicillic acid, mycophenolic acid, roquefortine C, and gliotoxin) in maize and wheat across two harvest seasons, 2014 and 2015. Evidence of mycotoxins’ persistence in Albanian cereals has demonstrated the existence of *Fusarium* mycotoxins, aflatoxin, and ochratoxin contamination [26,27,28].

## 2. Results

### 2.1. Method Validation Data

The validation was performed for all mycotoxins included in the article by spiking blank samples of maize and wheat with a mixed mycotoxin standard solution in acetonitrile at three levels: LOQ (25 µg/kg), 2LOQ (50 µg/kg), and 20LOQ (500 µg/kg). All data from measurements were evaluated, and recovery (Rec, %), repeatability (RDR_r_, %), and within-laboratory reproducibility (RDR_wR_, %) were determined as presented in Table 2. As the LOQ, the lowest validated level was chosen (25 µg/kg) and the LOD was determined as one-third of LOQ.

### 2.2. Incidence of Five Mycotoxins in Maize and Wheat

The results obtained in this study are shown in Table 3. The number and the percentage of positive samples; the mean value of positive samples; and the median, minimum, and maximum determined concentrations are presented. They comprise only the values of contaminated samples, while samples with values below the LOQ were considered to be 0.0 μg/kg and not included in the calculations. The maize samples from the 2014 season had the most significant incidence rate, with MPA being the most prevalent at 32.3%, followed by CPA at 12.9% and ROQ C at 6.5%. Penicillic acid and gliotoxin were not found in maize grain throughout both harvesting seasons. An analysis of five mycotoxins reveals distinct contamination patterns in two separate years of harvest. In 2014, CPA, MPA, and ROQ C were found, but in the 2015 harvest season, only MPA was discovered in maize samples. Mycophenolic acid exhibited the most significant concentration levels among the other mycotoxins investigated in our study, with a mean value of 1064 μg/kg and a range of 72.9–3447 μg/kg. Among the 31 maize samples from 2014, CPA was shown to have the second-highest occurrence rate at 12.9%.

The average concentration of CPA in these samples was 590.2 μg/kg, with a range of 189.2–868.9 μg/kg. ROQ C was identified as the third mycotoxin detected in maize, occurring with a relatively low frequency of 6.5%. The average concentration of ROQ C was 277.9 μg/kg, with a range of 124.6–431.2 μg/kg. The median results for positive samples were as follows: MPA had a concentration of 374.7 μg/kg, CPA had a concentration of 651.4 μg/kg, and ROQ C had a concentration of 277.9 μg/kg. The prevalence and frequency of MPA during the 2015 season exhibited a significantly high prevalence rate of 90% but a reduced frequency compared with the 2014 data, with an average value of 46.6 μg/kg and a range of 25.5–134.4 μg/kg—a co-occurrence of the mycotoxins MPA and ROQ C was detected in a single sample. The spread of pollution varies according to the area, primarily due to variances in the climate and the amount of precipitation. The maize samples (12) from the Elbasani region were non-contaminated. However, the maize grain from Fieri (18) had a contamination incidence of 7 out of 18 samples, with MPA detected in these samples, and 4 samples contaminated by CPA mycotoxin.

Similarly, 8 out of 11 samples of the maize commodity from the Korça region were contaminated with MPA, and 2 were contaminated by ROQ C. The Kruja region showed a similar pattern, with 8 out of 11 samples contaminated with MPA. *Fusarium* toxins were also observed with the subject mentioned [26].

## 3. Discussion

Fungal pathogens in cereal crops can significantly reduce output, with estimated losses ranging from 15% to 20%. In severe instances, these losses can escalate to as much as 50% [29]. Of the numerous mycotoxins, only around 20 are naturally occurring pollutants that pose a significant risk to food and feed safety. These mycotoxins, including aflatoxins, ochratoxin A, zearalenone, deoxynivalenol, and fumonisins, are of particular concern due to their frequent occurrence and possible toxicity [1]. Mycotoxins are classified into three main risk management categories on the basis of their toxicity and occurrence. The first category includes major mycotoxins that can potentially cause illness in humans or domestic animals and economic losses. The second category consists of minor mycotoxins shown to have toxicity or economic impact on a smaller scale. The third category includes mycotoxins of lesser importance, which have demonstrated toxicity but are not known to be associated with specific diseases. This is typically due to the uncommon occurrence of these foods [6]. Multiple species of *Penicillium* fungi, together with other mycotoxins such as cyclopiazonic acid, penicillic acid, mycophenolic acid, and roquefortine C, have been found in maize-based diets, including silage [11,30,31].

The two-year study indicates that infection in maize grains is more common than in wheat. Also, the contamination status was different among the two harvesting seasons, with a high infection rate in the samples from 2014 but not the same pattern in the following year, 2015. Considering the contamination status on the second crop commodity, the wheat, it was concluded that none of the five mycotoxins were found in this grain.

The presence of CPA has been documented in corn [32], cheese [33], and several types of animal feeds and feed ingredients [3,34]. While CPA is generally considered a non-significant mycotoxin, its exposure can be extensive [6]. Toxicological tests conducted on several species have shown that the primary organs affected are the liver, kidney, and digestive systems. The presence of CPA in broiler chickens has been found to affect their growth rate and feed intake [8]. The global presence of CPA is evidenced in various regions of the world (Table 4). In Asia, for instance, maize samples from Indonesia had CPA levels of LOD–9000 μg/kg [35], while poultry feed in the same region had levels ranging from 20 to 9220 μg/kg [36]. In India, feed contamination levels ranged from 400 to 12,000 μg/kg [34], and in Japan, maize samples showed relatively low contamination levels of 76 μg/kg [25]. The contamination levels of maize from the United States are reported to vary from 120 to 1820 μg/kg [37], with a limit of detection (LOD) of 2.771 μg/kg [32]. There is a lack of available data on the occurrence of CPA in Africa. However, research studies have revealed that in Mozambique, one out of every thirteen samples had a measured level of 606 μg/kg [38]. The contamination of maize indicates the presence of both CPA and AFs [27]. This discovery pertains to corn samples originating from the Korça area. CPA is recognized as a contributing factor in several chronic illnesses affecting both humans and animals. Significant findings will emerge from the link between CPA and AFs’ co-occurrence and the prevalence of chronic diseases in the human population of this region.

Maize with a high moisture content that has seen mold development has been discovered to have PA in very high quantities [48]. Information on maize harvested in Albania indicates a lack of application of quality standards, such as an indicator of penicillic acid’s occurrence in the analyzed maize samples [49]. Penicillic acid in feed has been reported in pig and chicken farms in Bulgaria and South Africa, with an average concentration of 904.9 μg/kg and a contamination range of 30–9220 μg/kg (Table 4) [39]. In a study conducted on maize from the United States, the average concentration of penicillic acid was 59 μg/kg, with a range of occurrence of 5–231 μg/kg [40].

ROQ C was detected in maize samples from the 2014 season, with a frequency of 6.5% in two out of thirteen samples. The concentration of ROQ C in these samples ranged from 124.6 to 431.1 μg/kg (Table 4). However, no contamination was observed in the samples evaluated from the 2015 season. The presence of ROQ C has also been documented in the feeds of many European Union nations, including the Czech Republic, Denmark, Hungary, and Germany. The contamination levels ranged from 1.3 to 14 μg/kg [41,43]. In Italy, the average contamination level in maize silage was 740 μg/kg with a range of LOD–32,000 μg/kg [44]. In the Netherlands, the contamination level in maize silage ranged from LOD to 3160 μg/kg [45]. In the United States, the contamination level in maize silage ranged from 20 to 1100 μg/kg [30]. Tangni and colleagues [18] discovered the existence of both mycotoxins in maize and grass silages given to dairy calves in Belgium. The levels of ROQ C contamination ranged from 459 to 1848 μg/kg, while MPA levels ranged from 4448 to 21,387 μg/kg for non-moldy and moldy silages, respectively [48]. The presence of ROQ C has been observed alongside MPA, CPA, and patulin in maize samples from Indonesia. The most significant occurrence was attributed to ROQ C toxin at a rate of 60%, followed by MPA at 42%, CPA at 37%, and PAT at 23% [35]. The occurrence of ROQ C in newly harvested maize for silage in the USA was detected in 30 out of 60 samples, with concentrations ranging from 20 to 1100 μg/kg [30].

MPA is synthesized by several strains of *P. roqueforti* and certain other *Penicillium* strains, notably *P. brevicompactum* and *P. paneum*. Mammals have a limited susceptibility to MPA toxicity. Administering a daily dosage of 30 μg/kg orally to rats leads to toxicity, causing anemia and death. MPA is commonly used to treat psoriasis; both MPA and its derivatives have demonstrated anticancer and immunosuppressive properties [20]. Out of the 31 samples, 10 were found to have MPA contamination. The concentration of MPA ranged from 72.9 to 3447 µg/kg, with an average value of 1064 μg/kg. Europe and the United States have comprehensive global statistics on the incidence of mycophenolic acid (Table 4). Mycophenolic acid and roquefortines are the most extensively researched chemicals generated from *Penicillium* in preserved goods. According to Gallo et al. [47], MPA is the most common fungal toxin found in silages after harvest, with concentrations exceeding 20,000 μg/kg. The mean level of roquefortine C contamination in silage was 778 μg/kg, while the mean level of mycophenolic acid contamination was 524 μg/kg. However, the highest levels of roquefortine C and mycophenolic acid contamination were 3160 μg/kg and 2630 μg/kg, respectively [50]. The presence of air leads to significantly elevated levels of MPA (9300 µg/kg) and ROC (26,000 µg/kg) in the surface layers of corn silages, as observed in a study publishing data from 2013 [18]. Studies have demonstrated that the tillage, cultivation, fertilization rate, and cultivar selection impact the occurrence of fungal infections in cereals. Nevertheless, the presence of these diseases is primarily influenced by the meteorological conditions that occur throughout the growth and development of plants [38].

### 3.1. Co-Occurrence with Fusarium, AFs, OTA, and Alternaria Toxins

The co-occurrence of CPA, PA, MPA, ROQ C, and GLI with *Fusarium* toxins, aflatoxins, ochratoxin A, and the *Alternaria* mycotoxins, referring to previously published data from the same grain samples, shows interesting perspectives [26,27,51]. The highest co-occurrence rate was revealed between CPA and *Fusarium* toxins, especially Fumonisin B1 and Fumonisin B2 [26]. Another close correlation was identified with AFs in contaminated maize samples [27]. Meanwhile, the co-occurrence between MPA and *Alternaria* toxins was found to be less present [51].

### 3.2. Climate in the Main Agricultural Regions of Albania

The country’s climate is characterized by average summer temperatures exceeding 20 °C, while winter temperatures range from −3 to 18 °C. According to the Köppen–Geiger climate classification, the western plain along the Mediterranean Sea has a hot summer Mediterranean climate (Csa) [50]. The mean annual temperature is 16.7 °C, with July having the highest temperature at 25.6 °C, and January being the coldest month with a temperature of 7.6 °C. The average annual precipitation is 803.1 mm. The Fieri district encompasses the primary agricultural area within this specific climate zone.

Conversely, the Elbasani area has a transitional microclimate that shifts towards a continental climate. It receives an average annual rainfall of 1007.0 mm; an unusual climatic event occurred in 2014 when it received high rainfall of 1282.0 mm throughout the summer. The Kruja region in Eastern Albania has a humid subtropical climate (Cfa) with an average annual temperature of 15 °C and annual precipitation of 1260.0 mm. On the other hand, the Korça plain, situated at an altitude of 800 m a.s.l, experiences a continental Mediterranean climate (Csb), with a yearly average temperature of 10.3 °C and precipitation of 695.5 mm, making it the driest region in the country [52].

## 4. Materials and Methods

### 4.1. Sample Collection

Plant material, including 51 maize and 70 wheat samples, were collected from different agricultural regions of Albania in two consecutive years, 2014 and 2015. Five mycotoxins (CPA, PA, MA, roquefortine C, and gliotoxin) were analyzed for their presence. The wheat and maize seed material was collected from small farms and warehouses during the two harvesting seasons. The location information with the respective latitude and longitude are presented in Table 5. The sampling procedure followed Commission Regulation [EC] No 401/2006 to ensure representative samples.

### 4.2. Standards and Chemicals

Individual standard solutions (100 µg/mL) for each mycotoxin were purchased from Romer Labs (Tulln, Austria). The stock and mixed working standard solution in acetonitrile were stored at −20 °C in amber glass vials. Chemical reagents of pure analytical grade, namely acetonitrile (AcCN), methanol (MeOH), acetic acid (Sigma-Aldrich, Steinheim, Germany), and ammonium acetate (Merck, Darmstadt, Germany), were purchased, while a Milli-Q system for preparing deionized water was used (Millipore, Bedford, MA, USA).

### 4.3. Sample Preparation

The simultaneous determination of mycotoxins consisted of extraction from the ground cereal samples and LC-MS/MS analysis [53]. The grains were ground to a particle size of 1 mm using a Retsch ZM 100 laboratory mill (Haan, Germany). A sample size of 10 g was extracted in a volume of 100 mL of an AcCN–deionized water mixture (84:16 *v*/*v*) and shaken for one hour using an IKA HS 501 digital linear shaker (IKA Labortechnik, Staufen, Germany). A 4 ml filtered extract was evaporated to dryness using a Syncore Polyvap system (Büchi, Flawil, Switzerland). The dry residue was reconstituted in 0.5 ml of MeOH/deionized water (80:20 *v*/*v*). Finally, 10 μl of the solution was injected into an UPLC-MS/MS system (Acquity UPLC H Class system) coupled to a triple quadrupole mass spectrometer (Xevo TQ MS) and a detection system equipped with electrospray ionization (ESI) interface and MassLynx software for data collection and processing (Waters, Milford, MA, USA). The vials were stored in the autosampler at 10 °C. For matrix-based calibration, appropriate amounts of the standard solutions were added to 4 ml aliquots of filtered extracts and prepared beside the samples.

### 4.4. LC-MS/MS Operation

A Zorbax Eclipse Plus C18 Rapid Resolution HD column (2.1 × 100 mm, 1.8 µm) from Agilent, was used for chromatographic separation. Mycotoxin separation was performed using a mixture of Solvents A (deionized water, 2.5 mM ammonium acetate, and 0.5% acetic acid) and B (MeOH, 2.5 mM ammonium acetate, and 0.5% acetic acid) in a gradient condition with the mobile phase at a fixed flow rate of 0.3 ml min^−1^ and the temperature of the column at 40 °C. Multiple reaction monitoring (MRM) mode was employed for MS/MS analysis. Specific MS/MS parameters related to the determined mycotoxins (retention times, ionization mode, and monitored transitions) are given in Table 6. The LOD and LOQ for the analyzed mycotoxins were 8.3 µg kg^−1^ and 25 µg kg^−1^.

### 4.5. Statistical Analysis

Mycotoxin concentrations were calculated using two replicates. The incidence of positive samples and the mean, median, maximum, and minimum values were determined.

## 5. Conclusions

This study presents findings on five unregulated mycotoxins in maize and wheat grain collected over two harvesting seasons, 2014–2015. Three out of five studied mycotoxins, cyclopiazonic acid, mycophenolic acid, and roquefortine C, revealed their presence in maize from 2014; meanwhile, during the 2015 harvest season, the MPA was the only detected mycotoxin. This mycotoxin was the most prevalent in both harvesting seasons and had the maximum occurrence. In conclusion, their contamination levels and presence in maize are of high concern compared with wheat crops. While exposure evaluations for people and animals do not currently consider these mycotoxins, it is crucial to investigate their presence in grains due to the potential for combined harmful effects when co-exposed with other controlled mycotoxins. The contamination patterns in maize samples from the Kruja and Korça regions showed a significant occurrence rate, which was associated with the climatic features of these locations.

## Figures and Tables

**Table 2:** Three-level spiking, repeatability (RSDr), intra-laboratory reproducibility (RSD_wR_) and the recovery rate in maize and wheat.

Analytes	Spiking Level (µg/kg)	RSDr (%)	RSD_wR_ (%)	Rec (%)	RSDr (%)	RSD_wR_ (%)	Rec (%)
		Maize			Wheat		
PA	25	4.0	11.7	109	11.3	12.9	98
	50	3.2	10.2	101	8.0	11.9	108
	500	1.3	7.9	104	2.0	4.0	103
MPA	25	6.0	12.4	95	4.5	11.2	103
	50	5.0	10.2	102	2.0	9.3	105
	500	1.8	8.0	100	1.7	8.9	104
ROQ C	25	11.6	17.6	106	13.9	15.5	101
	50	7.8	14.5	101	8.1	13.1	97
	500	3.0	12.4	91	1.7	8.5	96
CPA	25	13.5	21.6	102	9.2	12.4	115
	50	8.1	20.7	105	5.0	17.8	97
	500	4.3	14.6	104	3.5	15.3	100
GLI	100	6.2	11.9	113	7.7	11.2	97
	200	8.2	10.0	99	6.1	10.8	102
	2000	3.0	8.5	103	3.1	7.2	98

Legend: PA—penicillic acid; MPA—mycophenolic acid; ROQ C—roquefortine C; CPA—cyclopiazonic acid; GLI—gliotoxin; RDRr—repeatability; RDR_wR_—within-laboratory reproducibility; Rec—recovery.

**Table 3:** Maize contamination during the harvesting seasons of 2014 and 2015 (μg/kg).

2014					
Parameter	Cyclopiazonic Acid	Penicillic Acid	Mycophenolic Acid	Roquefortine C	Gliotoxin
Analyzed samples	31	31	31	31	31
Positive samples	4	0	10	2	0
Incidence (%)	12.9	0.0	32.3	6.5	0.0
Min	189.1	0.0	72.9	124.6	0.0
Max	868.9	0.0	3447	431.2	0.0
Mean ^a^	590.2	0.0	1064	277.9	0.0
Median ^a^	651.4	0.0	374.7	277.9	0.0
Mean ^b^	76.2	0.0	343.2	17.9	0.0
2015					
Analyzed samples	20	20	20	20	21
Positive samples	0	0	18.0	0	0
Incidence (%)	0.0	0.0	90.0	0.0	0.0
Min	0.0	0.0	25.5	0.0	0.0
Max	0.0	0.0	134.4	0.0	0.0
Mean ^a^	0.0	0.0	46.6	0.0	0.0
Median ^a^	0.0	0.0	37.3	0.0	0.0
Mean ^b^	0.0	0.0	31.9	0.0	0.0

^a^ Calculated by including only positive samples. ^b^ Calculated by considering the values of negative samples to be 0.0 μg/kg.

**Table 4:** Worldwide incidence of the studied mycotoxins in corn, feed, and maize silage.

	Country	Commodity	Incidence of Positive Samples	Mean (μg/kg)	Range (μg/kg)	Reference
Cyclopiazonic acid (CPA)	Indonesia	Feed	1/1	6000	6000	[35]
		Feed	21/26	–	LOD–9000	
	Indonesia/Australia	Poultry feed	19/26	2117	30–9220	[36]
	USA	Feed	33/38	390	120–1820	[37]
	India	Feed	10/26	–	400–12,000	[34]
	USA	Maize	23/45	–	LOD–2771	[32]
	Japan	Maize	1/6	76	76	[25]
	Mozambique	Feed	1/13	606		[38]
Penicillic acid (PA)	Bulgaria/South Africa	Feed	23/25	904.9	30–9220	[39]
	USA	Maize	7/20	59	5–231	[40]
Roquefortine C (ROQ C)	Czech Republic, Denmark, Hungary	Feed	4/82	4.6	1.3–14	[41]
	USA	Maize silage	30/60	–	20–1100	[42]
	Germany	Maize silage	12/12	17,000	700–36,000	[43]
	Italy	Maize silage	10/196	740	LOD–32,000	[44]
	Netherlands	Maize silage		778	LOD–3160	[45]
Mycophenolic acid (MPA)	USA	Maize silage	16/60	–	80–600	[42]
	Germany	Maize silage	38/135	690	20–23,000	[46]
	Italy	Maize silage	16/196	1760	LOD–48,000	[44]
	Netherlands	Maize silage		524	LOD–2630	[47]

LOD—limit of detection.

**Table 5:** Locations (latitude/longitude) of the sampling sites in this study (decimal degrees).

	Region	Site	Latitude	Longitude	North/East
1	Fieri	F1	41.03941	19.61639	41°02’21’’/19°36’59’’
2		F2	41.05049	19.63315	41°03’01’’/19°37’58’’
3		F3	40.81566	19.64542	40°48’56’’/19°38’43’’
4		F4	40.95259	19.58174	40°57’09’’/19°34’54’’
5		F5	40.94701	19.66672	40°56’49’’/19°40’00’’
6		F6	40.59749	19.57707	40°35’51’’/19°34’37’’
7		F7	40.76345	19.43551	40°45’48’’/19°26’08’’
8		F8	40.74414	19.49718	40°44’38’’/19°29’49’’
9		F9	40.60508	19.61362	40°36’18’’/19°36’49’’
10		F10	40.76236	19.51895	40°45’44’’/19°31’08’’
11	Elbasan	E1	40.97925	20.00892	40°58’45’’/20°00’32’’
12		E2	40.92760	20.00727	40°55’39’’/20°00’26’’
13		E3	41.02681	19.98925	41°01’36’’/19°59’21’’
14		E4	41.00312	20.01798	41°00’11’’/20°01’05’’
15	Korça	Ko1	40.74278	20.71750	40°44’34’’/20°43’03’’
16		Ko2	40.78767	20.71908	40°47’15’’/20°43’09’’
17		Ko3	40.70656	20.79229	40°42’23’’/20°47’32’’
18		Ko4	40.74301	20.77187	40°44’34’’/20°46’19’’
19		Ko5	40.79772	20.776875	40°47’51’’/20°46’36’’
20		Ko6	40.703721	20.73280	40°42’13’’/20°43’58’’
21	Kruja	K1	41.43284	19.71172	41°25’58’’/19°42’42’’
22		K2	41.47498	19.65192	41°28’29’’/19°39’07’’
23		K3	41.51103	19.69971	41°30’39’’/19°41’58’’
24		K4	41.55797	19.69142	41°33’28’’/19°41’29’’
25		K5	41.604533	19.67878	41°36’16’’/19°40’43’’

**Table 6:** Mass spectrometric detection parameters: retention time, precursor, quantifier, and qualifier ion for ROQ C, GLI, MPA, PA, and CPA.

Mycotoxin	Abbrev.	Ionization Mode	Retention Time (min)	Precursor Ion (*m*/*z*)	Quantifier Ion (*m*/*z*)	Qualifier Ion (*m*/*z*)
Roquefortine C	ROQ C	ESI +	9.10	390.3	193.0	322.2
Gliotoxin	GLI	ESI −	7.25	325.1	261.1	243.1
Penicillic acid	PA	ESI +	4.10	171.1	97.0	124.9
Mycophenolic acid	MPA	ESI +	10.2	321.2	207.0	159.0
Cyclopiazonic acid	CPA	ESI +	12.5	337.2	196.1	182.1

## Data Availability

The original contributions presented in the study are included in the article, further inquiries can be directed to the corresponding author/s.
